# Active Polymer Gel Actuators

**DOI:** 10.3390/ijms11010052

**Published:** 2010-01-05

**Authors:** Shingo Maeda, Yusuke Hara, Ryo Yoshida, Shuji Hashimoto

**Affiliations:** 1 Department of Applied Physics, Waseda University, 3-4-1 Okubo Shinjuku-ku, Tokyo, 169-8555, Japan; E-Mails: yhara@shalab.phys.waseda.ac.jp (Y.H.); shuji@waseda.jp (S.H.); 2 Department of Materials Engineering, Graduate School of Engineering, The University of Tokyo, 7-3-1 Hongo, Bunkyo-ku, Tokyo, 135-8656, Japan; E-Mail: ryo@cross.t.u-tokyo.ac.jp (R.Y.)

**Keywords:** polymer gel, polymer actuator, oscillating reaction

## Abstract

Many kinds of stimuli-responsive polymer and gels have been developed and applied to biomimetic actuators or artificial muscles. Electroactive polymers that change shape when stimulated electrically seem to be particularly promising. In all cases, however, the mechanical motion is driven by external stimuli, for example, reversing the direction of electric field. On the other hand, many living organisms can generate an autonomous motion without external driving stimuli like self-beating of heart muscles. Here we show a novel biomimetic gel actuator that can walk spontaneously with a worm-like motion without switching of external stimuli. The self-oscillating motion is produced by dissipating chemical energy of oscillating reaction. Although the gel is completely composed of synthetic polymer, it shows autonomous motion as if it were alive.

## 1. Introduction

Stimuli-responsive polymers and gels that swell or shrink in response to environmental changes [1–5] have been studied by many researchers. The importance of these characteristics has been recognized from not only scientific but also engineering points of view. Nowadays, certain devices made of electro-active polymers have been developed [6,7]. They are expected to act as soft actuators because not only they are light, soft and flexible materials, but they can also generate biomimetic motion without using mechanical parts such as motors and drive shafts, gears, *etc*. In previous decades, the chemomechanical system of transforming chemical energy to mechanical energy using the reversible conformation changes of collagen fibers was demonstrated [8]. Also, since volume phase transition of polymer gels was found by Tanaka [9], many kinds of applications such as a drug delivery system [10], robotic hands [11], and matter transporting device [12], have been proposed in various fields. The phase transition of polymer gel is induced by hydrogen bonds, coulomb, hydrophobic and van der Waals interactions. Thus, by changing the external physicochemical conditions, these applications can be controlled. For major example, poly(*N*-isopropylacrylamide) (PNIPAAm), which is a thermo-sensitive polymer, undergoes a discontinuous volume change. In application, a microfluidic device using MEMS technology and PNIPAAm, could adsorb proteins from solution and release them due to the adsorption change of PNIPAAm by controlling resistive heating [13].

However, these systems require complex and fabricated circuits or external control devices because the resulting mechanical motion is driven by on–off switching of external signals. On the other hand, in biological systems, there are several autonomic phenomena exhibiting spontaneous motion without any external stimuli such as peristaltic motion, self-oscillation, *etc*. If such a system is to be achieved in an artificial system, a novel actuation device that does not depend on external control would be expected. Thus, devices mirroring living organisms could be created without electronic wiring. There are currently only a few reports of the incorporation of such autonomous devices into artificial systems. As an attempt using cardiac muscle cells and synthetic polymers, Whitesides and co-workers have demonstrated a self-walking bioactuator [14]. Although utilization of biopolymer or cell system is one possible way, our aim is to realize a completely artificial system.

**Figure 1.**
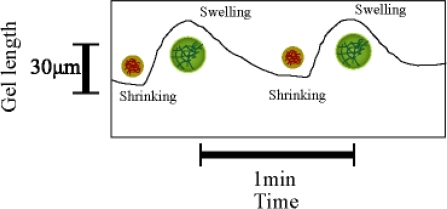
Conventional self-oscillating gel.

In this review, we introduce active polymer gel actuators. Gels exhibit a unique capability of undergoing spontaneous volume changes in response to oscillatory chemical reaction. For example, gels can undergo rhythmic swelling and shrinking in response to variations in the pH of the surrounding solution caused by an oscillating reaction. The pH oscillations were generated by the Landolt reaction [15] or by an enzymatic reaction [16]. Periodical volume changes were also observed in a pH responsive gel, which exhibited bistability with diffusion of a reactive substrate into the gel [17]. In all these studies, the cause of the complex behaviors was outside of the gels. Furthermore, the chemical reactions were carried out under nonequilibrium conditions provided by precisely controlled continuous-flow stirred tank reactors [15,17]. This means that the condition around the gel is not stationary. A self-oscillating gel that moved under stationary conditions is only known [18]. The self motion of the gel is produced by dissipating chemical energy of the oscillatory Belousov-Zhabotinsky (BZ) reaction [19]. The BZ reaction is the most commonly known oscillating reactions. The overall process is the oxidation of an organic substrate such as citric or malonic acid by an oxidizing agent (bromate) in the presence of a metal catalyst under acidic condition. Metal ions or metal complexes with high redox potentials, such as cerium ion, ferroin, or ruthenium tris(2,2′-bipyridine) are widely used as catalysts. In the course of the reaction, the catalyst ion periodically changes its charge number to oscillate between the oxidized and reduced states for several hours as long as the substrate exists. In an unstirred solution, there are chemical waves and spatial pattern formations in the reaction process. The use of the gels in the BZ reaction has been so far confined to eliminating hydrodynamic convection disturbing reaction diffusion patterns [20] and localizing the BZ reaction in a restricted region [21]. In this systems, the structure of gels do not change by the BZ reaction because the interaction between the gel structure and the reaction diffusion system is negligibly small. It was reported that liner polymers and gels undergo a slight spontaneous structure changes [19,22,23]. In oscillating gel system, the polymer networks itself takes part in the BZ reaction, because the metal catalyst is covalently bounded to the polymer chain. As a result, the reaction medium can change in time and space. In other words, the BZ reaction induces the structural changes. When the redox state of the metal catalyst moiety in the gel changed, the solubility of the polymer chain changed as well. As a result, the change in the osmotic pressure inside the gel causes the swelling or shrinking of the polymer gel. The displacement of the self-oscillating gel is several dozen micrometers [19] as shown in Figure 1. However, the mechanical displacement of the gel was too small to design the gel actuator. Therefore, in order to construct gel actuators, we should improve the displacement of the gel greatly.

## 2. Design of Self-walking Gel

**Figure 2.**
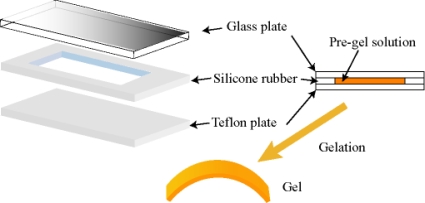
Preparation of the poly[NIPAAm-co-Ru(bpy)_3_-co-AMPS] gel membrane undergoing anisotropic contraction. Reproduced from [25].

For realizing the large deformation of the gel, we adopted the method of introducing a gradient structure into the gel. In order to make the gradient structure in the gel, we utilized the hydrophobic interaction between the Ru(bpy)_3_^2+^ moiety and the casting mold during the polymerization. As a result, the large deformation of the self-oscillating gel was achieved [24]. During the polymerization, the monomer solution faces two different surfaces of plates: a hydrophilic glass surface and a hydrophobic, Teflon surface. as shown in Figure 2.

**Figure 3.**
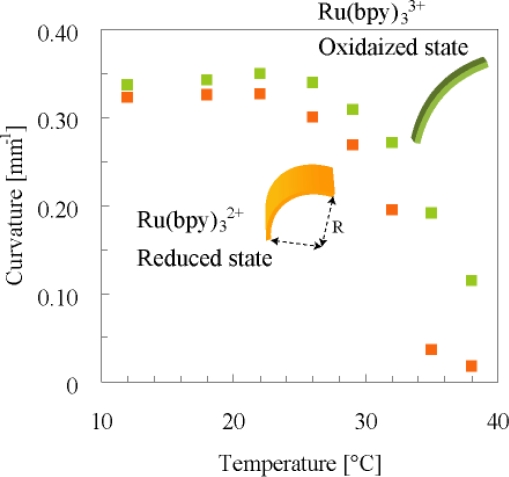
Equilibrium swelling ratio expressed as curvature of the poly(NIPAAm-co-Ru(bpy)_3_-co-AMPS) gel with gradient structure. Closed square: Ce_2_(SO_4_)_3_ = 0.005 M and HNO_3_ = 0.894 M; Open square: Ce(SO_4_)_2_ = 0.005 M and HNO_3_ = 0.894 M. The curvature is defined as 1/R. Reproduced from [25].

Since Ru(bpy)_3_^2+^ monomer is hydrophobic, it easily migrates to the Teflon surface side. As a result, a uniform distribution in the direction of the thickness is formed for the component, and the resulting gel has a gradient distribution for the content of each component in the polymer network. Thus, the hydrophilic AMPS component at the glass side was higher than that at the Teflon side. In contrast, the hydrophobic Ru(bpy)_3_^2+^ moiety at the Teflon side was higher than that at the glass side. Therefore, as for the gel at the AMPS rich side, the swelling ration was higher than that at the opposite side (at the Ru(bpy)_3_^2+^ rich side). Consequently the gel in water bends to the direction of the surface which was faced to Teflon plate during polymerization. Figure 3 shows the curvature changes of the poly(NIPAAm-co-Ru(bpy)_3_-co-AMPS) gel strip as a function of temperature under the different conditions of the reduced Ru(II) and oxidized Ru(III) states. To maintain the equilibrium condition of Ru(II) or Ru(III) states, the whole of gel was homogeneously reduced or oxidized forcibly by using reducing and oxidizing agent [Ce(III) and Ce(IV)], respectively. As a result of the characteristics of the thermosensitive NIPAAm component, the curvature decreases with an increase in the temperature. The curvature in the oxidized state was larger than that in the reduced state all over the temperature range. This is because when the hydrophilicity of the polymer increases, the gel expands in the oxidized state. From the deviation of the curvature in the Ru(II) and the Ru(III) states, we expected that the gel caused the periodical bending-stretching motion induced by the BZ reaction at constant temperatures.

**Figure 4.**
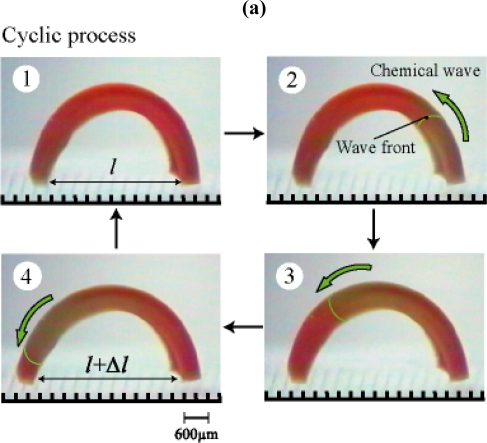

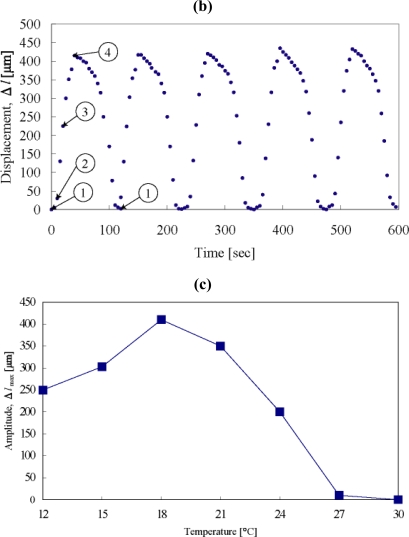
(a) Equilibrium swelling ratio expressed as curvature of the poly(NIPAAm-co-Ru(bpy)_3_-co-AMPS) gel strip in cerium sulfate solutions as a function of temperature. Closed square: Ce_2_(SO_4_)_3_ =0.005 M and HNO_3_ = 0.894 M; Open square: Ce(SO_4_)_2_ = 0.005 M and HNO_3_ = 0.894 M. The curvature is defined as 1/R. (b) Oscillating profiles of the bending-stretching motion for the gel. *l* is the direct distance between two edges of the curved gel strip at reduced state. Δ*l* is the displacement of the direct distance when chemical wave propagates in the gel. (c) Dependence of amplitude (the maximum of displacement, Δ*l*_max_) on temperature. Reproduced from [25].

Figure 4(a) shows the cyclic changes of bending-stretching motion of the gel strip in an aqueous solution containing the three reactants of the BZ reaction (malonic acid, sodium bromate and nitric acid) at constant temperatures. Since the gel size is larger than chemical wavelength, homogeneous redox changes induced forcedly by using the oxidizing and reducing agents as shown in Figure 2 do not occur. The chemical wave evolves in the gel, and it propagates in the direction of the length at constant speed from one edge attaching the substrate to the other edge. With the propagation of the chemical wave, the distance between the two edges of the gel changes periodically because the spontaneous bending and stretching motion occurs [Figure 4(b)]. While the chemical wave exists in the gel (1→4), the gel stretches. After that, during the reduced state until the next wave appears (4→1), the gel bends. As shown in Figure 4(c), the amplitude of the mechanical oscillation (Δ*l*_max_) changes with temperature because the difference in swelling ratio between reduced state and oxidized state depends on temperature (c.f. Figure 3). We can see that there is the optimum temperature (18 °C) at which the amplitude becomes the maximum. This temperature does not necessarily coincide with the optimum temperature estimated from Figure 3. This is because Figure 3 indicates the comparison of equilibrium redox states in different solutions from the BZ substrate solution. And also, in the case of Figure 4(c), the oscillation period changes with temperature, which affects the dynamic process of mechanical oscillation. Anyway, the following experiment was conducted at 18 °C.

**Figure 5.**
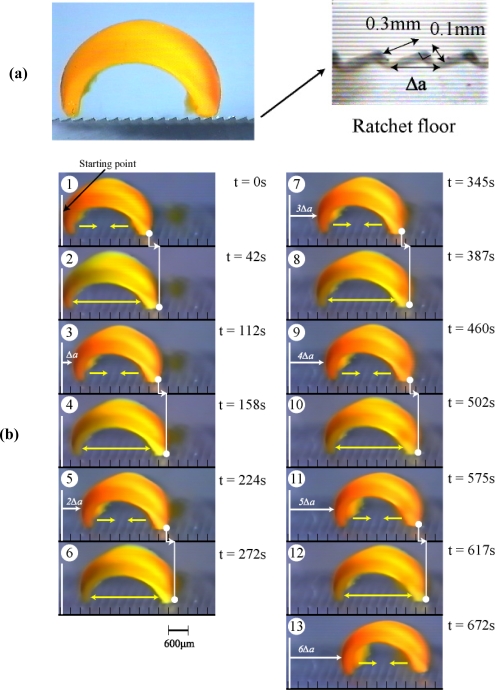
(a) Surface structure of the ratchet floor made of acrylic sheet. Δ*a* stands for the interval of the ratchet teeth. (b) Time course of self-walking motion of the gel actuator (odd number: bending process at the reduced state, even number: stretching process with propagation of chemical wave). During stretching process, the front edge can slide forward on the floor, but the rear edge is prevented from sliding backwards. Oppositely, during bending process, the front edge is prevented from backwards while the rear edge can slide forward. This action is repeated spontaneously, and as a result, the gel walks forward. In one period of the oscillation, the gel can take a step forward by Δ*a.* Outer solution: 62.5 mM malonic acid, 84 mM sodium bromate, 0.894 M nitric acid, 18 °C. Reproduced from [25].

In order to convert the bending and stretching changes to one-directional motion, we employed a ratchet mechanism. A ratchet floor with asymmetrical surface structure made of acrylic sheet was fabricated [Figure 5(a)]. On the ratchet floor, the gel repeatedly bends and stretches autonomously, but backwards sliding is prevented by the teeth of the ratchet. As a result, the gel can move forward. Figure 5(b) shows successive profiles of the “self-walking” motion of the gel like a looper in the BZ substrate solution under constant temperature [25]. The period of chemical oscillation was about 112 sec, and the walking velocity of the gel actuator was about 170 μm/min. Since the oscillating period and the propagating velocity of chemical wave change with concentration of substrates in the outer solution, the walking velocity of the gel can be controlled.

## 3. Design of Peristaltic Motion of a Polymer Gel

**Figure 6.**
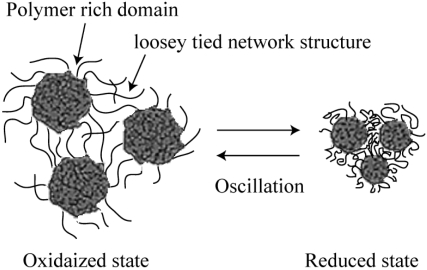
Illustration of the porous gel.

However, it was difficult to observe the peristaltic motion coupled with the chemical wave directly because the mechanical oscillation was too small in comparison with the gel size. Theoretical studies have predicted the occurrence of peristaltic motion within the gel [26,27]. In previous report, the peristaltic motion of the gel has been indirectly evaluated from its color tones [28]. However, there is no report for the direct observation of the macroscopic peristaltic motion of the gel coupled with the BZ reaction. Recently, we succeeded for the first time in observing the peristaltic motion of the gel [29] directly by utilizing a novel gel with a porous structure. We focus on the kinetics of the polymer gel. The network motion of the gel as given by Tanaka, Hocker and Benedek [30] is:

(1) $$\frac{\partial \vec{u}}{\partial t}=\frac{K+\mu /3}{f}grad(div\vec{u})+\frac{\mu }{f}\Delta \vec{u}$$

where Δ denotes the Laplacian and $\vec{u}(\vec{r},t)$ is the displacement vector that represents the displacement of a point in the network. *K*, *μ* and *f* are the osmotic bulk modulus, shear modulus of polymer gel and friction coefficient between network and fluid medium, respectively. In the case of a radial deformation (1) becomes:

(2) $$\begin{array}{l} \frac{\partial u}{\partial t}=D_{c}\frac{\partial }{\partial r}\left(\frac{\partial u}{\partial r}+2\frac{u}{r}\right) \end{array}$$

with

$$D_{c}=\frac{K+4/3\mu }{f}$$

where *D_c_* is the diffusion coefficient of the polymer gel. If *D_c_* has a high value, the response of the gel is fast, as shown in Equation (2). In general, the degree of the response of hydro gels composed of chemically cross-linked polymer networks is low because the polymer chains are molecularly restricted by a large number of cross-links. There are remarkable difference of swelling ratio between the reduced Ru(II) state and oxidized Ru(III) state in the poly[NIPAAm-co-Ru(bpy)_3_] gel at the equilibrium swelling state. But actually, the volume oscillation coupled with the redox oscillation of the ruthenium catalyst moiety due to the BZ reaction is very small. The rate of the redox reaction of the Ru moiety is significantly faster than that of swelling-deswelling of the gel in the equilibrium condition such as above mentioned. Therefore, the poly[NIPAAm-*co*-Ru(bpy)_3_] self-oscillating gel generated the small mechanical oscillation. In order to produce the large mechanical oscillation in comparison with the gel size, the self-oscillating gel has to response firstly to the rate of the BZ reaction. For realizing this purpose, we prepared the microphase-separated self-oscillating gel. That is because that, in the previous work, it was reported that the NIPAAm gel with micro scale phase separation underwent quick response [31]. By preparing NIPAAm gel above the lower critical solution temperature (LCST), the network structure becomes inhomogeneity due to the LCST nature of the NIPAAm component. As a result, the NIPAAm gel forms a porous structure that consists of two regions: one is polymer rich domains, and the other is aggregations in the matrix of a loosely tied network structure. Consequently, rich domains inside the gel clump or loose rapidly because of an effluent pathway of water due to the porous structure, as shown in Figure 6. However, the micro phase separation in the gel strongly depends on the methods of gel preparation. Therefore, the control of the phase separation was too difficult by selecting the synthesis temperature. In order to control the micro scale phase separation into the self-oscillating gel, we synthesized the gel under the water-methanol mixture solution by utilizing the hydrophobic casting mold. Generally, in the mixed solvent of water and methanol, the LCST of aqueous PNIPAM solutions shifts to lower temperature [32,33]. So, it is assumed that the micro phase separated structure was introduced inside the gel.

**Figure 7.**
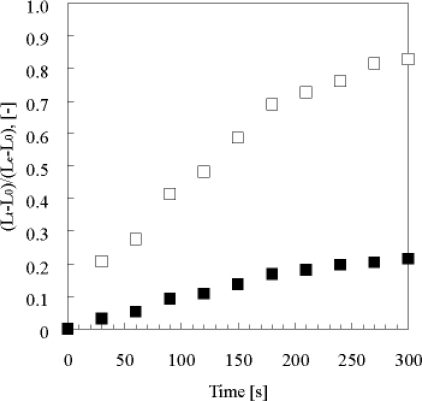
Relative swelling of microphase-separated self-oscillating gel and poly(NIPAAm-co-Ru(bpy)_3_) gels, (*L_t_* – *L*_0_)/(*L_e_* – *L*_0_), in the solution of 5 mM Ce(SO_4_)_2_, 0.894 M HNO_3_ at 18 °C as functions of the time *t* elapsing after changing the solution. *L_t_*, *L*_0_ and *L_e_*, are the lengths of the gel at *t* = t, initial state and equilibrium state. (□) microphase-separated self-oscillating gel; (▪) poly(NIPAAm-*co*-Ru(bpy)_3_) gel. Reproduced from [29].

**Figure 8.**
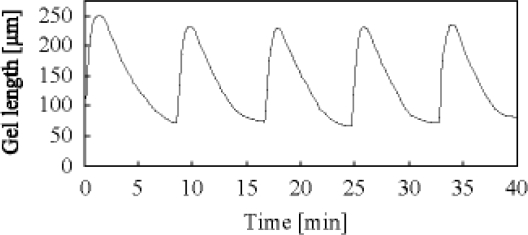
Oscillating profile of cubic gel. Cubic gel (each side length is about 0.5 mm) was immersed in 1 mL of the mixture solution of the BZ substrates (62.5 mM malonic acid, 84 mM sodium bromate, 0.894 M nitric acid, 18 °C). Reproduced from [29].

As shown in Figure 7, the swelling speed of the microphase-separated self-oscillating gel was faster than that of the poly[NIPAAm-*co*-Ru(bpy)_3_] gel at 18 °C. This result indicated that the swelling dynamics of the microphase-separated self-oscillating gel is different from the poly[NIPAAm-co-Ru(bpy)_3_] gel. The data supported that the time scale of the swelling kinetics and the chemical reaction matched. This result is significantly importance to cause large deformation of the gel by utilizing the BZ reaction. Next, we prepared the cubic gel of which size was smaller enough than the wavelength of the chemical wave. Within the miniature gel, the redox change homogeneously occurred without evolution of chemical waves. As for the miniature gel, the oscillating profiles of the redox changes as well as the swelling-deswelling changes were analyzed by using the image-processing method. Figure 8 shows the self-oscillating behavior of the cubic gel in the aqueous solution containing the three reactants of the BZ reaction (malonic acid, sodium bromate and nitric acid) at the constant temperature. The displacement of the mechanical oscillation was around 130 μm. The amplitude of the volume oscillation for the microphase-separated self-oscillating gel is about ten times as large as that for the poly[NIPAAm-*co*-Ru(bpy)_3_] gel. This result indicated that the large mechanical oscillation of the gel required the rapid response to the change in the redox state of the metal catalyst induced by the BZ reaction. From this result, it is expected that the gel of which size is larger enough than the wavelength of the chemical wave undergoes periodical peristaltic motion when the redox state of the Ru(bpy)_3_ moiety in the gel periodically change by the BZ reaction at the constant temperature.

**Figure 9.**
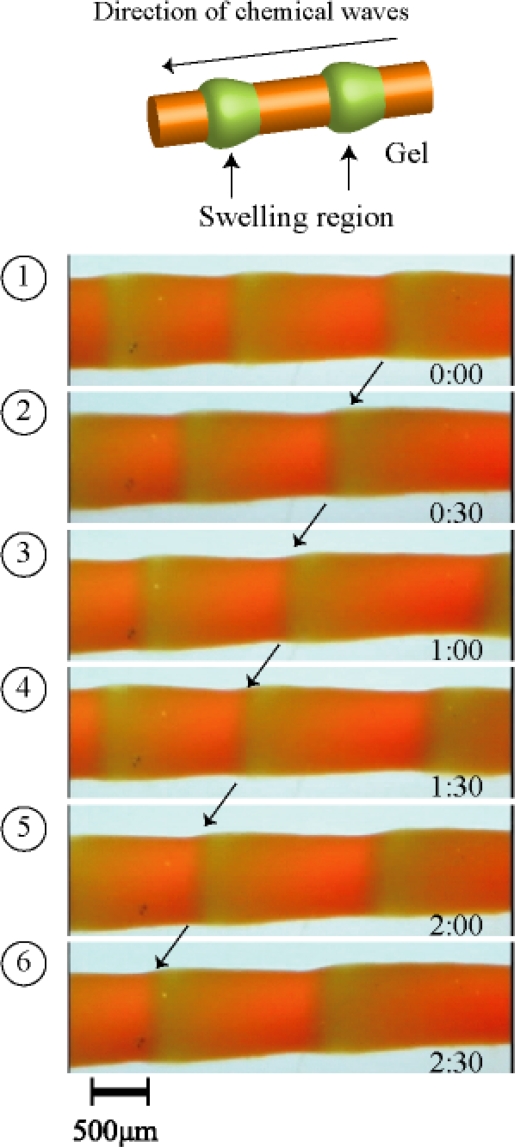
Time course of peristaltic motion of microphase-separated self-oscillating gel in 8 mL of the mixture solution of the BZ substrates (62.5 mM malonic acid, 84 mM sodium bromate, 0.894 M nitric acid, 18 °C). The green and orange colors correspond to the oxidized and reduced state of Ru moiety in the gel, respectively. Reproduced from [29].

**Figure 10.**
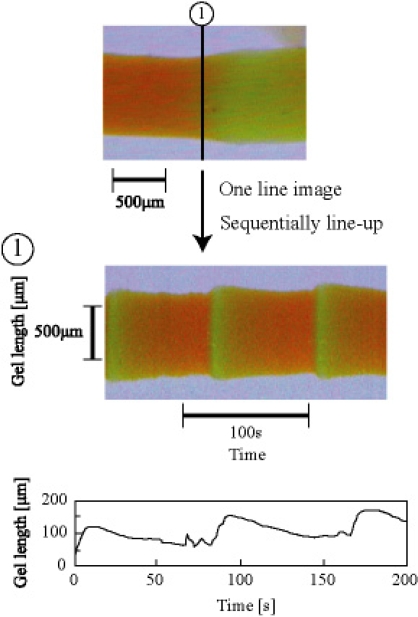
Spatio-temporal pattern of gel oscillation. Reproduced from [29].

Figure 9 shows the periodical peristaltic motion of the gel driven by the chemical waves of the BZ reaction. We first succeed in observing the periodical peristaltic motion of the gel directly. With the propagation of the chemical waves, the local swelling regions propagated in the gel. This is the first visual evidence of the peristaltic motion of the gel in the macroscopic scale. The shape change of the gel was in excellent agreement with the theory [26,27]. The aspects of the volume change of the gel followed the reaction diffusion dynamics. The chemical wave speed of the BZ reaction was approximately 14.0–30.0 μm/sec in the gel. Figure 10 shows the spatio-temporal diagram constructed from the sequential images. The width of the gel changed shape periodically with about 150 μm. As shown in Figure 8, the change in the width of the gel increased c.a. 20% from the initial width of the gel. The period of the volume oscillation was about 86 sec.

## 4. Matter Transport

**Figure 11.**
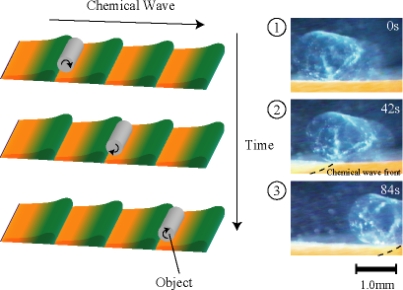
Schematic illustration of the matter transport using peristaltic motion of the gel. Reproduced from [29].

Furthermore, we succeeded in conveying the object by utilizing the peristaltic motion of the gel. We set the cylindrical polyacrylamide gel as the object on the rectangular microphase-separated self-oscillating gel in the aqueous solution containing the three reactants of the BZ reaction. Figure 11 shows the illustration of matter transport. The peristaltic surface of the gel pushed and carried the object by rotating it in one direction at about 40 μm/sec with the chemical wave propagation as shown in Figure 9. The gel conveyer carried the object with millimeter order autonomously. It is assumed that the peristaltic motion of the gel can be controllable by changing the concentration of the BZ substrates because the spatiotemporal dynamic pattern changes with changing the outer solution.

## 5. Conclusions

Now, the actuation of the gel is operated under non-physiological environment where the three substrates of the BZ reaction coexist. If the actuation can drive under biological conditions, realization of novel intelligent bio-machines or devices without relying on the electricity would be expected. Therefore, we have been challenging the modification of the molecular structure of the gel in order to cause self-oscillation under physiological condition, that is, only in the presence of bio-related organic acids such as citric acid [34–36]. Moreover, recently, by optimizing the polymer design, we succeeded in modifying the driving speed of the gel in 0.5 Hz [37]. The maximum frequency (0.5 Hz) of the novel gel was 20 times as large as that for the conventional-type self-oscillating gel. The autonomous peristaltic motion of the gel would create a new design method for matter transport, micro or nano devices, *etc*.
